# Magnetic Properties of Collagen–Chitosan Hybrid Materials with Immobilized Superparamagnetic Iron Oxide Nanoparticles (SPIONs)

**DOI:** 10.3390/ma14247652

**Published:** 2021-12-12

**Authors:** Sylwia Fiejdasz, Adriana Gilarska, Tomasz Strączek, Maria Nowakowska, Czesław Kapusta

**Affiliations:** 1 Department of Solid State Physics, Faculty of Physics and Applied Computer Science, AGH University of Science and Technology, Mickiewicza 30, 30-059 Krakow, Poland; kapusta@agh.edu.pl; 2 Faculty of Chemistry, Jagiellonian University, Gronostajowa 2, 30-387 Krakow, Poland; nowakows@chemia.uj.edu.pl; 3 Department of Robotics and Mechatronics, Faculty of Mechanical Engineering and Robotics, AGH University of Science and Technology, Mickiewicza 30, 30-059 Krakow, Poland; tsr@agh.edu.pl

**Keywords:** magnetic hybrid materials, magnetic properties, vibrating sample magnetometry (VSM), blocking temperature, SPIONs

## Abstract

The paper presents results of our studies on hybrid materials based on polymers of natural origin containing superparamagnetic iron oxide nanoparticles (SPIONs). Such nanoparticles, coated with the chitosan derivative, were immobilized in a chitosan-collagen hydrogel matrix by crosslinking with genipin. Three types of biopolymer matrices of different collagen-to-chitosan ratios were studied. A thorough magnetic characterization was performed, including magnetic susceptibility, magnetization, and hysteresis loop measurements in a temperature range of 4 K to 300 K and a magnetic field induction up to 8 Tesla. The effect of SPION immobilization and material composition on the magnetic properties of the hybrids was investigated. The results showed that hybrid materials with covalently bounded SPIONs preserved the superparamagnetic character of SPIONs and exhibited promising magnetic properties, which are important for their potential applications.

## 1. Introduction

Hydrogel materials, defined as three-dimensional crosslinked polymer networks [1], have a broad spectrum of applications in the biomedical field; e.g., in targeted drug delivery [2], biosensors [3], pharmaceuticals [4], and tissue engineering [5]. This is due to their resemblance to the natural extracellular matrix (ECM), hydrated environment, and tunable physicochemical properties [6]. Hydrogels can be obtained using synthetic polymers or those of natural origin. The popular examples of hydrogels prepared from synthetic polymers are based on polyethylene glycol (PEG) [7], polylactic acid (PLA) [8], polyglycolic acid (PGA) [9], polyacrylates [10], and poly(NIPAM) [11]. Hydrogels can be also based on biopolymers such as collagen, chitosan [12], alginate [13], dextran [14], or hyaluronic acid [15].

Incorporation of nanoparticles in polymer matrices has a significant impact on their properties; e.g., it can enhance their electrical conductivity, chemical resistance, and mechanical properties [16,17,18]. Materials with magnetic functionality are nowadays receiving considerable attention. This is why magnetic nanoparticles are often used to create hybrid materials [19,20]. Magnetic hydrogels have been developed for a broad spectrum of applications; e.g., in drug delivery [21], image enhancement [22], or remote-controlled actuators [23]. Magnetic hybrid hydrogels are composed of a polymer matrix with an incorporated magnetic phase, usually in the form of magnetite, maghemite, or ferrite nanoparticles [24,25]. Superparamagnetic iron oxide nanoparticles (SPIONs) are used for that purpose due to their features such as superparamagnetism and responsiveness to external magnetic fields [26]. There are two methods of magnetic nanoparticle entrapment in the matrix—by physical interactions or by permanent immobilization via chemical bonds [6]. The properties of magnetic hydrogels (including magnetic response) are affected by many factors; e.g., the type of hydrogel and nanoparticles used, the degree of dispersion of NPs within material, the strength of inter-particle interactions, and nanoparticle concentration and size [27,28]. 

In the current report, we present a detailed analysis of magnetic properties of hybrid hydrogel materials with covalently bonded SPIONs. The hybrid materials were prepared using three types of polymer matrices with different collagen-to-chitosan ratios. The influence of nanoparticle immobilization in a hydrogel matrix on the magnetism of the systems obtained was investigated. Another aspect studied was the impact of the hybrids’ composition on their magnetic properties. The measurements were carried out with vibrating sample magnetometry, and the comparison between samples was made using values of saturation magnetization, coercivity, magnetic susceptibility, superparamagnetic blocking temperature, and differential susceptibility. Nanoparticle-only material in the form of surface-coated SPIONs was also studied for comparison purposes.

## 2. Materials and Methods

### 2.1. Materials

We used collagen type I rat tail (3.52 mg/mL solution, BD Biosciences), chitosan (low molecular weight, Sigma-Aldrich), genipin (Challenge Bioproducts Co., 98%), acetic acid (Chempur), ethanol 96% (POOH), iron(III) chloride hexahydrate and iron(II) chloride tetrahydrate (Sigma-Aldrich), and ammonia (25% solution, puriss. p.a) (Sigma)

### 2.2. Synthesis

- Hydrogels

Hydrogel matrices based on biopolymers: collagen and chitosan (ColCh) were obtained by mixing the appropriate amounts of: 2 wt % chitosan solution in 1% acetic acid, collagen stock solution, and 100 μL of 0.1% genipin solution in ethanol per 0.5 mL of sol. Volume ratios of polymers used (collagen:chitosan) varied between the samples, and were equal to 0:100, 25:75, and 50:50 for ColCh 0:100, ColCh 25:75, and ColCh 50:50, respectively. The mixtures were incubated at 37 °C until gels were formed.

- Superparamagnetic iron oxide nanoparticles (SPIONS)

Nanoparticles were synthesized according to the procedure developed by us and described in [29]. Firstly, iron chlorides in molar ratio Fe(III):Fe(II) = 2:1 were weighed and then dissolved in aqueous solution of cationic derivative of chitosan (50 mL, 1 g/L). In the next step, the reaction mixture was sonicated for 10 min in a thermostatic bath (20 °C) in the presence of argon. It was followed by dropwise addition of 5 mL of ammonia (5 M) and further sonication for 30 min. After the synthesis, the obtained nanoparticles were purified using magnetic filtration.

- Hybrid materials

Hybrids were obtained by introducing SPIONs in form of the aqueous dispersion (75 μL, 235 μg/mL) to the collagen–chitosan sols (0.5 mL) prepared as described in Section 2.2 (Hydrogels). The formulations were vortexed and incubated at 37 °C until gel formation was achieved. Finally, the subsequent hybrid materials were obtained: ColCh 0:100 S1, ColCh 25:75 S1, and ColCh 50:50 S1.

### 2.3. Methods/Apparatus

Determination of the magnetic properties of materials was done in their dry state using the vibrating sample magnetometer option of a physical property measurement system (Quantum Design, San Diego, CA, USA) equipped with a superconducting 9 Tesla magnet. Hysteresis loops were measured at selected temperatures in the range of 4 K to 300 K and magnetic field induction ranging from −8 to +8 Tesla.

The differential susceptibility was calculated as a derivative of specific magnetization using OriginPro 9.0 SR2 data-analysis software (OriginLab, Northampton, MA, USA). The documentation for the program stated that the derivative in a given point is calculated as mean of slopes between the neighboring points.

The derivative function applied to discrete data points can therefore be written:

$$f^{\prime }\left(x_{i}\right)=\frac{1}{2}\left(\frac{y_{i+1}-y_{i}}{x_{i+1}-x_{i}}+\frac{y_{i}-y_{i-1}}{x_{i}-x_{i-1}}\right)$$

## 3. Results

### 3.1. Hybrid Materials Obtained

Superparamagnetic iron oxide nanoparticles (SPIONs) with chitosan coatings were prepared according to the method developed by us and described earlier [29]. Small objects with hydrodynamic diameters of about 100 nm (as determined by DLS) and a magnetic core of 10 nm (as determined by TEM) [30] having inverse spinel structure of magnetite or maghemite (determined by XRD), and relaxational character, typical for superparamagnets (as observed using Mössbauer technique), were obtained. Their low-temperature Mössbauer spectra closely resembled those of maghemite [31]. Hybrid magnetic materials were prepared by introducing the SPIONs surface-coated with cationic derivative of chitosan to the hydrogels at the stage of synthesis and crosslinking the system with genipin [32]. Three hybrid materials differing in the composition of polymer matrix, but having the same concentration of SPIONs (S1), were fabricated (ColCh 0:100 S1, ColCh 25:75 S1, and ColCh 50:50 S1). The materials obtained were proved to be structurally stable by turbidity measurements. Their microstructures were characterized using SEM observations. A good/homogenous distribution of magnetic nanoparticles inside the hybrid was confirmed using STEM technique. Rheological studies indicated that the values of storage modulus were dependent on the composition of the hydrogel matrix (storage modulus increases with chitosan content) and the presence of magnetic nanoparticles in their structure. Additionally, magnetic force microscopy (MFM) measurements confirmed that magnetic nanoparticles were well dispersed within hybrid materials. A scanning transmission electron microscopy (STEM) study showed that the SPION nanoparticles tend to align along the collagen fibers.

### 3.2. Magnetic Characterization

The preliminary study of the hybrids carried out earlier confirmed their magnetic properties and the homogenic distribution of magnetic domains in the materials’ volume. The observed differences in properties of these materials motivated us to obtain a deeper insight into their magnetic properties in a wider range of temperatures (4–300 K). The factors that were considered included: SPION immobilization in hybrid material (comparison of magnetic properties of SPION nanoparticles and hybrids), the effect of matrix composition/properties, and SPION concentration.

#### 3.2.1. Hysteresis Loops/Coercive Field/Ms

**Figure 1:**
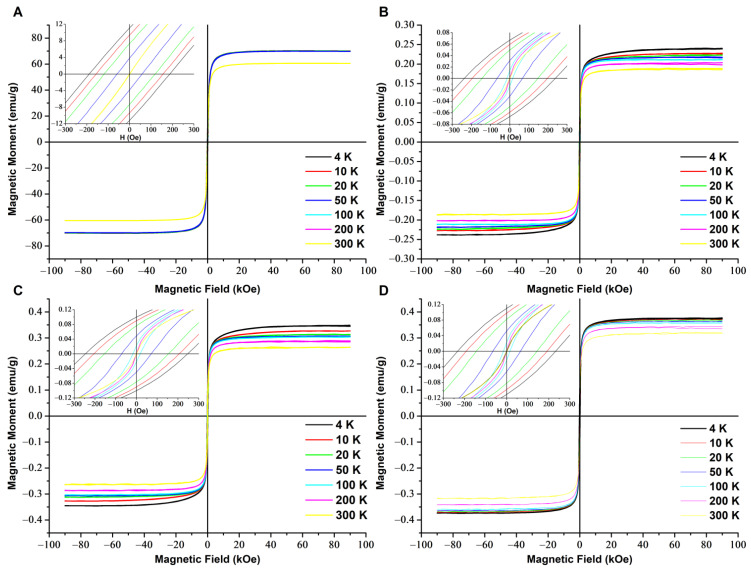
Magnetization curves measured for SPIONs (**A**) and hybrids: ColCh 0:100 S1 (**B**), 25:75 S1 (**C**), and 50:50 S1 (**D**). The insets show an enlarged region at the origin.

The magnetic properties of the hybrids containing SPION nanoparticles (ColCh 0:100 S1, ColCh 25:75 S1, and ColCh 50:50 S1) were measured in the 4−300 K temperature range using a vibrating sample magnetometer (VSM). The magnetic nanoparticles (SPIONs) were also tested for comparison. Magnetization curves as a function of the applied magnetic field are shown in Figure 1.

**Figure 2:**
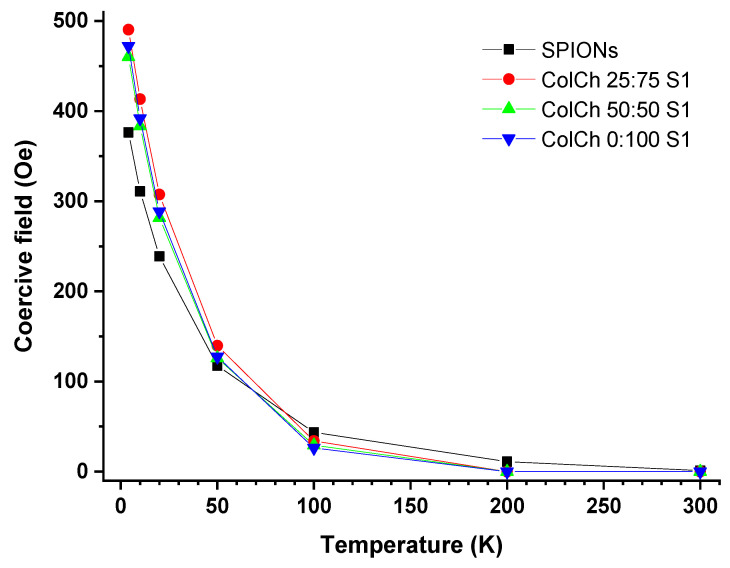
Temperature dependence of the values of the coercive field, Hc, for the materials studied.

The temperature dependence of the coercive field, H_c_, is presented in Figure 2. The magnetic hysteresis loops for all the hybrid materials exhibited a similar shape and vanishing coercivity at 200 K and 300 K. These results indicated that in the hybrid materials, the nanoparticles were in a superparamagnetic state at T ≥ 200 K. For the SPION sample containing magnetic nanoparticles only (Figure 1A), the coercive field was still nonzero at 200 K, which meant that its blocking temperature was considerably higher than that characteristic of the hybrid materials. Surprisingly, the coercive field at low temperatures was larger for the hybrids, and at 4 K it was 30% higher than for the SPIONs. A similar effect was also observed for polymer nanocomposites with magnetic fillers [33]. This finding was attributed there to the specific organization of magnetic fillers with chain formation, branching, and clustering of chains, resulting in large anisotropy leading to enhanced collective magnetic response. The coercive fields measured for our hybrid composites at 100 K, slightly below their blocking temperatures, did not differ within the error margin of 5 Oe, and they amounted to 26 Oe, 29 Oe, and 34 Oe for the ColCh 0:100 S1, 25:75 S1, and 50:50 S1 samples, respectively.

The much lower saturation magnetization of the hybrids than that of the SPION-only material reflected the amount of SPIONs taken for preparation of the hybrids. The differences in the saturation magnetization values between the hybrids were attributed to different water losses when drying the samples. It also indicated that the amount of water absorbed in the hybrids was larger for the higher collagen content.

**Figure 3:**
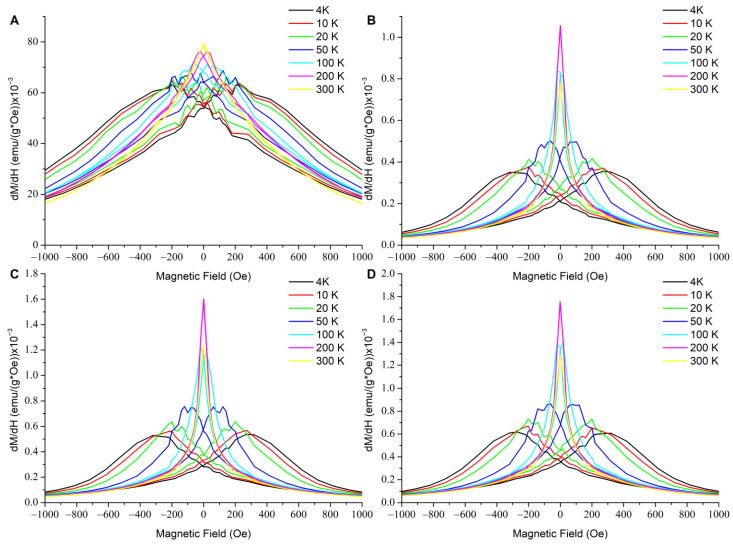
Derivatives of the magnetization curves (i.e., differential susceptibilities) for the materials studied: SPIONs (**A**), ColCh 0:100 S1 (**B**), 25:75 S1 (**C**), and 50:50 S1 (**D**) at different temperatures.

**Figure 4:**
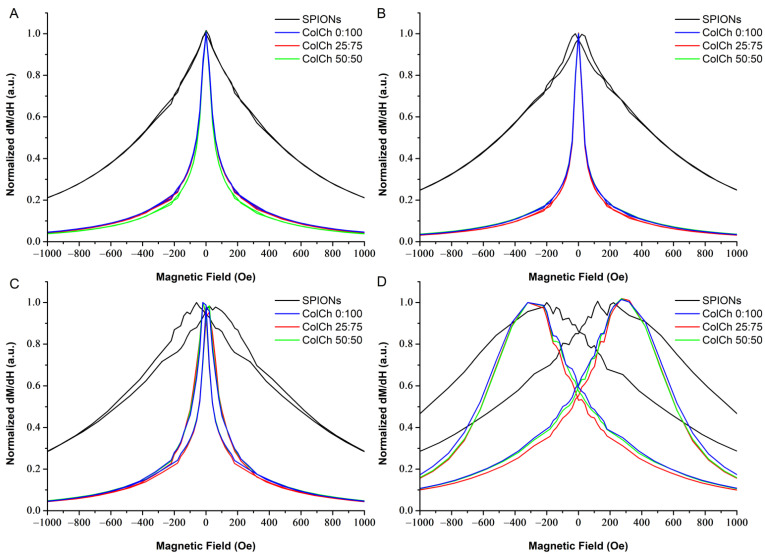
Normalized derivative of magnetization (normalized differential susceptibility) in function of the magnetic field at different temperatures: 300 K (**A**), 200 K (**B**), 100 K (**C**), and 4 K (**D**) for SPIONs and hybrid materials.

In order to observe the magnetization process closer, the derivatives of the magnetization loops (differential susceptibility) were taken; these are presented in Figure 3 for individual samples and in Figure 4 for all the samples at selected/defined temperatures. The up and down magnetizing curves do not coincide at 4 K and 100 K for any of the materials studied. For the hybrids, they coincide at 200 K and 300 K, which corresponds to the lack of coercivity, and indicates their superparamagnetic state at these temperatures. For the SPIONs, they still do not coincide at 200 K, which confirmes their much higher blocking temperature. These results correspond with the observation of a vanishing coercivity in the magnetization loops presented in Figure 1. It is worth noting that the differential susceptibility curves of the hybrids become narrower upon going from 300 K to 200 K, and then they are much broader at 4 K. This is clearly visible in Figure 3 and Figure 4. 

This narrowing effect reflected increased low field susceptibility upon approaching the blocking temperature. This indicated that nanoparticles characterized by their blocking temperature slightly below the room temperature would be optimal; e.g., for magnetic control of their distribution with low fields on preparation of hybrid materials. They would have the highest low field response and preserve superparamagnetic properties at room temperature.

The saturation magnetization (Ms) at 300 K for ColCh 0:100 S1, ColCh 25:75 S1, and ColCh 50:50 S1 amounts to 0.20, 0.26, and 0.35 emu/g, respectively. These values are significantly lower than the saturation magnetization for SPIONs only (60.1 emu/g); i.e., maghemite nanoparticles in chitosan shells. This could be explained by considering that the saturation magnetization of magnetic hybrid materials was proportional to the product of concentration of magnetic nanoparticles and their saturation magnetization [34,35]. As the samples studied were subsequently dried for magnetization measurements, the difference in saturation magnetization was attributed to different water losses in the samples. The water loss determined for the ColCh 0:100 S1, 25:75 S1, and 50:50 S1 samples was 98.21%, 98.62%, and 98.97%, respectively. Recalculation of the saturation magnetization values of dry samples to their masses resulted in respective values related to the SPION nanoparticles’ maghemite phase in these materials of 81.8, 81.7, and 82.3 emu/g.

To have a deeper insight into that issue/matter, measurements of hybrids with 20 times higher nanoparticle content, corresponding to 0.6 mg Fe per mL of hydrogel (named ColCh 0:100 S10, ColCh 25:75 S10, and ColCh 50:50 S10, respectively) were carried out. The magnetic properties of these hybrids were measured at 300 K using VSM. The obtained values of Ms at 300 K for ColCh 0:100 S10, ColCh 25:75 S10, and ColCh 50:50 S10 ranged between 4 and 7 emu/g, with the same tendency as for the hybrids of a 20 times lower nanoparticle content. The hysteresis loops showed a vanishing coercivity, revealing that these much more concentrated magnetic hybrids were also superparamagnetic at room temperature, as the hybrid with lower content of SPIONs was.

Phenomena to be considered included dipolar interactions or more complex interfacial interactions related to the distribution of the chainlike structures containing SPIONs in the hydrogel matrix, size of SPION aggregates, and SPIONs binding with polymeric matrix. Analysis of the STEM images [32] showed that in the samples containing collagen, the SPIONs formed aggregates that were smaller than in the chitosan, and tended to organize along the collagen fibers. The corresponding difference was reflected in the differential susceptibility (Figure 4), where its curve was narrower (showing a stronger field dependence of the low field susceptibility) for the collagen containing hybrids, and most clearly appeared for the highest collagen content (50:50, green line) at 300 K. The effect of polymer matrix on magnetic properties of nanocomposites with Fe_3_O_4_ was also investigated in [36]. It was concluded in that study that interfacial interactions played an important role, and affected the magnetic properties of the hybrids. The correlation between rheological and magnetic properties was also noted.

#### 3.2.2. ZFC/FC Curves (Magnetic Susceptibility, Blocking Temperature)

**Figure 5:**
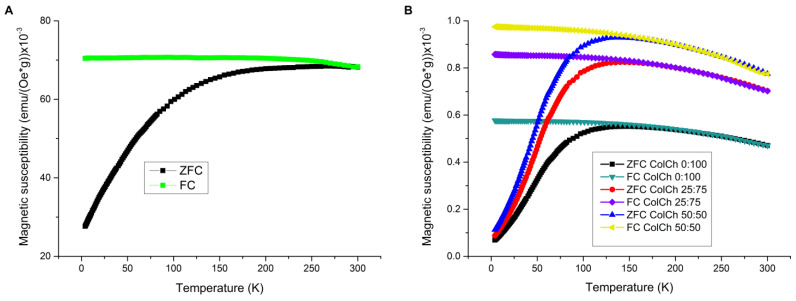
ZFC/FC curves measured for SPIONs (**A**) and hybrids (**B**).

Temperature dependences of the magnetic susceptibility, χ, for SPIONs and hybrid materials were also determined. The zero field cooled curves (ZFC) and field cooled curves (FC) measured in a magnetic field of 100 Oe are shown in Figure 5. The shape of the ZFC/FC curves is similar for all the hybrid materials studied. In the case of SPIONs, ZFC is typical for strongly interacting systems, consisting of a broad, rounded peak having a maximum close to RT. When interactions between magnetic nanoparticles are relatively strong, the magnetization values of the low-temperature part of the FC curve are closer to those at the maximum in ZFC, which was the case for SPIONs. The blocking temperatures, T_B_, for the hybrids studied were estimated as corresponding to the maximum of their ZFC curve. Their values amounted to 150 K, 140 K, and 135 K for the ColCh 0:100 S1, ColCh 25:75 S1, and ColCh 50:50 S1 samples, respectively; i.e., the larger the chitosan content, the higher the blocking temperature. This also concerned the SPION-only sample containing concentrated nanoparticles with chitosan coatings, which exhibited the highest T_B_ of 270 K. These results also confirmed that the nanoparticles dispersed in the hydrogels were superparamagnetic at room temperature, and the blocking temperature for hybrid materials was lower than for the SPION-only sample. This could be explained by considering the much weaker interparticle interactions of SPIONs dispersed in hydrogels in comparison with the nanoparticle-only system. In the case of material containing only SPIONs, in which the concentration of nanoparticles was much higher than in the hybrids, the dipolar interactions may have affected the blocking temperature, shifting it towards higher temperatures [37,38]. That effect was also observed by Socolovsky [39] for magnetite NPs embedded in a PVA matrix.

When comparing the obtained values of magnetic susceptibility for SPIONs and hybrids, a difference of two orders of magnitude could be observed. This was in line with the above discussed concentration issues and the resultant interparticle distance and interactions. The same tendency regarding the magnetic susceptibility of hybrid materials was noticed, as in the case of saturation magnetization. Here, small differences between materials in χ value also were observed, and as in Ms, they were rising with collagen content, which confirmed the effect of the matrix composition.

**Figure 6:**
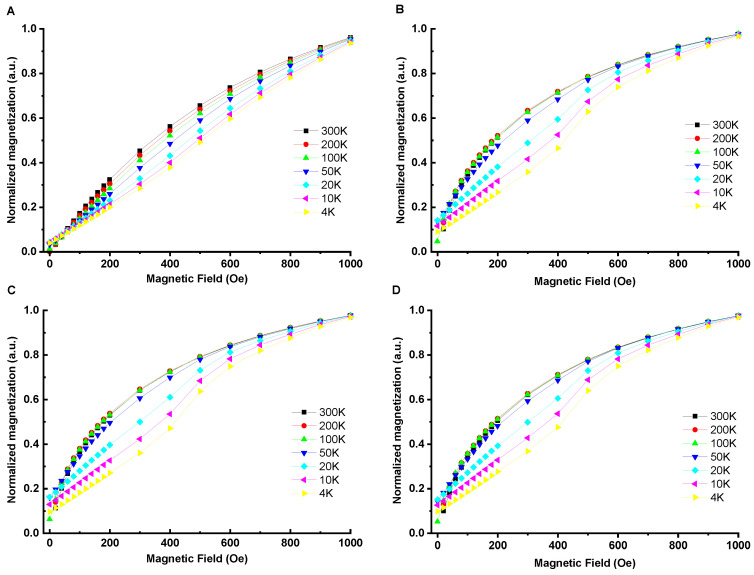
Normalized initial magnetization curves for the materials studied: SPIONs (**A**), ColCh 0:100 S1 (**B**), 25:75 S1 (**C**), and 50:50 S1 (**D**) taken at different temperatures.

A comparison of the initial magnetization curves of the hybrid and the SPION-only materials (Figure 6) showed that the former exhibited a clear negative curvature at low temperatures. This pointed towards different mechanisms of the magnetization reversal process in these systems, effected by strong interparticle interactions. In the SPION sample, the nanoparticles were close to each other, while in the hybrids, the distances between them were much larger, which limited their interactions. The higher relative value of the low-temperature ZFC susceptibility for the SPION sample was consistent with its lower coercive field (Figure 2). This indicated a possible domination of a reverse-domain-nucleation-like mechanism in the SPION material, consistent with magnetic dipole interactions between closely located nanoparticles, preferring antiparallel alignment of their moments and supporting them to act like reverse domains. For hybrids, a domain-wall-pinning-like mechanism dominated, which was also consistent with their coercivity being higher than those of SPIONs.

## 4. Conclusions

The results of our study confirmed that one can obtain the magnetic hybrid materials by incorporating superparamagnetic iron oxide nanoparticles (SPIONs) coated with a chitosan derivative in the structure of a hydrogel prepared from natural polymers. As shown by thorough magnetic characterization in a wide range of temperatures (4−300 K), the hybrid materials preserved superparamagnetic properties of the SPIONs above 200 K. It was observed that the blocking temperatures of hybrids were lower than those of the SPION-only material, and decreased with increasing collagen content.

The hydrogel matrix composition influenced the saturation magnetization values, which was attributed to different water content and its corresponding loss when drying the material. In our previous study [32], it was also observed that SPIONs formed aggregates of different sizes and align in chainlike structures along collagen fibers. This affected their differential susceptibility, generally exhibiting the highest low-field response to the magnetic field around the blocking temperature. The differential susceptibility revealed much stronger field dependence for the hybrids than for the SPIONs.

A concave shape of initial magnetization curves of the hybrids and the SPIONs suggested the contribution of a pinning-type mechanism of the magnetization reversal process. The effect differed between various samples, and was more pronounced for the hybrids than for the SPIONs, in which the nucleation type mechanism originating from dipolar interactions between less-separated nanoparticles could be more effective.

The materials used in this study for preparation of hydrogel matrices were of natural origin and proved to be biocompatible. We have previously shown that hydrogels prepared from biopolymers such as collagen and chitosan and crosslinked with genipin are promising materials for biomedical applications [5]. By adding SPIONs, we obtained hybrid materials with magnetic functionality, which broadens the area of their possible applications. They were proved to be structurally stable, which shows that SPIONs can be effectively immobilized in a hydrogel matrix, avoiding phase separation [32]. We have shown that magnetic nanoparticles immobilized in hybrids can preserve their superparamagnetic character above 200 K. Our study indicated that the magnetic hybrids we developed are promising materials for future biomedical and technological applications. A possible way of exploiting their potential would be aligning collagen fibers in the applied magnetic fields with the help of magnetic nanoparticles grouping along them, thus producing scaffolds for tissue regeneration. This, however, requires further studies.

## Data Availability

Not applicable.
